# DNMT 1 maintains hypermethylation of CAG promoter specific region and prevents expression of exogenous gene in fat-1 transgenic sheep

**DOI:** 10.1371/journal.pone.0171442

**Published:** 2017-02-03

**Authors:** Chunrong Yang, Xueying Shang, Lei Cheng, Lei Yang, Xuefei Liu, Chunling Bai, Zhuying Wei, Jinlian Hua, Guangpeng Li

**Affiliations:** 1 Research Center for Laboratory Animal Science, Inner Mongolia University, Hohhot, China; 2 College of Animal Science and Technology, Huazhong Agricultural University, Wuhan, China; 3 College of Animal Science and Technology, Northwest A&F University, Yangling, China; 4 College of Veterinary Medicine, Northwest A&F University, Yangling, China; Qingdao Agricultural University, CHINA

## Abstract

Methylation is an important issue in gene expression regulation and also in the fields of genetics and reproduction. In this study, we created fat-1 transgenic sheep, investigated the fine-mapping and the modulatory mechanisms of promoter methylation. Sheep fetal fibroblasts were transfected by pCAG-fat1-IRES-EGFP. Monoclonal cell line was screened as nuclear donor and carried out nuclear transfer (441 transgenic cloned embryos, 52 synchronism recipient sheep). Six offsprings were obtained. Expressions of exogenous genes fat-1 and EGFP were detectable in 10 examined tissues and upregulated omega-3 fatty acid content. Interestingly, more or less EGFP negative cells were detectable in the positive transgenic fetal skin cells. EGFP negative and positive cells were sorted by flow cytometry, and their methylation status in the whole promoter region (1701 nt) were investigated by bisulphate sequencing. The fine-mapping of methylation in CAG promoter were proposed. The results suggested that exogenous gene expression was determined by the methylation status from 721–1346 nt and modulated by methylation levels at 101, 108 and 115 nt sites in CAG promoter. To clarify the regulatory mechanism of methylation, examination of four DNA methyltransferases (DNMTs) demonstrated that hypermethylation of CAG promoter is mainly maintained by DNMT 1 in EGFP negative cells. Furthermore, investigation of the cell surface antigen CD34, CD45 and CD166 indicated that EGFP positive and negative cells belong to different types. The present study systematically clarified methylation status of CAG promoter in transgenic sheep and regulatory mechanism, which will provide research strategies for gene expression regulation in transgenic animals.

## Introduction

Transgenic animal technology is one of the fastest growing biotechnology areas. It is used to integrate exogenous genes into the animal genome by genetic engineering technology so that these genes can be inherited and expressed in offspring. Transgenic animal technology is in the process of revolutionizing the way we domesticate the livestock [1]. Further studies will allow transgenic technology to explore gene function, animal genetic improvement, bioreactor, animal disease model, and organ transplantation [2]. *In vitro* transfection of cultured differentiated cells combined with nuclear transfer is currently the most effective procedure producing transgenic animals. Improvements in the technology of producing transgenic farm animals are highly desirable because the economic advantages thus gained may benefit both biotechnology and basic research. The main barrier in transgenic animal production lies in identifying more efficient systems of transgenic delivery and better mechanisms to optimize the regulation of transgenic expression levels [3].

Epigenetic regulation of gene transcription involves DNA methylation, histone modification, chromatin remodelling, etc. DNA methylation is now recognized to be a chief contributor to the stability of gene expression states. Specifically, DNA methylation establishes a silent chromatin state by collaborating with proteins that modify nucleosomes [4]. Genes can be transcribed from methylation-free promoters even though adjacent transcribed and nontranscribed regions are extensively methylated [5]. DNA methylation in mammals is a post-replication modification that is predominantly found in cytosines of the dinucleotide sequence CpG [6].

DNA methyltransferase (DNMT) family catalyze the transfer of a methyl group to DNA. DNA methylation serves a wide variety of biological functions. All the known DNA methyltransferases use S-adenosyl methionine (SAM) as the methyl donor. Three active DNMTs have been identified in mammals. They are named DNMT 1, DNMT 3A, and DNMT 3B. A fourth enzyme previously known as DNMT 2 is not a DNA methyltransferase. DNMT 3L is a protein closely related to DNMT 3A and DNMT 3B in structure and critical for DNA methylation, but appears to be inactive on its own [7].

The fat-1 gene from the roundworm *Caenorhabditis elegans* encodes an omega-3 fatty-acid desaturase enzyme that converts omega-6 to omega-3 fatty acids and which is absent in most animals, including mammals [8]. Since then, fat-1 transgenic animals are rapidly prosperous, especially emerging as a new tool for studying the benefits of omega-3 fatty acids and the molecular mechanisms of their action in mice [9–11]. Up to now, except for mouse, fat-1 transgenic animals has been carried out in rat [12], pig [13, 14], cattle [15–17], sheep [18]. In the previous study, authors concluded CAG promoter, not CMV promoter was suitable for generation of fat-1 transgenic sheep. However, fine mechanism remains unclear.

In the present study, sheep fetal fibroblasts from a Day-55 Dubo sheep fetus were isolated, and transfected with fat-1 overexpression plasmid. We carried out nuclear transfer and embryo implantation using the positive monoclonal and untransfected cells. Skin cells from positive fat-1 transgenic fetuses were subjected to single cell sorting. Positive and negative cells from positive transgenic animals were employed for investigating fine-mapping of the whole CAG promoter methylation by bisulfite sequencing, DNMTs expressions, and cell types. The results clarified the regulatory mechanisms of the exogenous expression in details.

## Materials and methods

### Ethics statement

All animal work was conducted in accordance with the Guide for the Care and Use of Laboratory Animals, the Chinese Veterinary Medical Association (CVMA), and approved by the Institutional Animal Care and Use Committee at Inner Mongolia University, which is fully accredited by Association for Assessment and Accreditation of Laboratory Animal Care International. The experiments were complied with the Chinese Code of Practice for the Care and Use of Animals for Scientific Purposes, including conditions for animal welfare and handling prior to sacrifice.

Non-steroidal anti-inflammatory drug (FLUNDAN MEGLUMINE INJECTION, Hebei Yuanzheng Pharmaceutical Co., Ltd, China) was used to prevent wound inflammation and pain for three days, after the caesarean section. The sampled ewes were also monitored their health status, which included body temperature, pulse measurement for heart rate and chewing activity. There was no serious adverse events occurred for these ewes. All the sampled individuals (embryos, fetus, and newborns) were euthanized by electrical stunning followed by exsanguination, if necessary.

### Transfection of sheep fetal fibroblast cells and screening of the fat-1 transfected cells

Expression vector pCAG-fat-1-IRES-EGFP (abbreviation pfat-1) has been described in our previous study [18]. Briefly, neomycin (G418) gene cassette is used for positive screening of the fat-1 transgenic cells. Enhanced green fluorescent protein (EGFP) gene serves as a marker, which is co-expression with fat-1 gene. Internal ribosome entry site (IRES) has been widely used to co-express heterologous gene products by a message from a single promoter. IRES maintains independent expression of fat-1 and EGFP in the present study.

Sheep fetal fibroblasts derived from a Day 55 Dubo sheep fetus were cultured in DMEM/F12 (1:1) containing 10% fetal bovine serum (FBS). pfat-1 expression vector was transfected into sheep fetal fibroblast cells in a 24-well plate, with 750 ng of plasmid DNA and 5 μl Lipofectamine LTX per well. Fresh DMEM/F12 (1:1) supplemented with 15% FBS was added at 8 h post transfection. After 48 h culture, cells were divided into lower density (1:40 dilution) and screened with 800 μg/ml G418. At about 14 days post-transfection, G418-resistant and EGFP positive monoclonal cells were transferred into a 48-well plate. After several subcultures, stable G418-resistant and EGFP positive clones were obtained. These clones were subjected to PCR examination for fat-1 genomic integration analysis and RT-PCR investigation for fat-1 mRNA expression assay.

### Transgenic cloned embryo and embryo transfer

Generation of transgenic cloned embryos and embryo transfer were performed as previously described [18]. Briefly, transgenic cloned embryos were obtained by somatic nuclear transfer, subsequently these embryos were transferred by surgery to synchronized recipient. Non-transgenic sheep fetal fibroblasts from same fetus carried out somatic nuclear transfer and embryo implantation, we got the fetuses as control. Pregnancy was detected by ultrasonography (Ausonics, Australia) on approximately 70 days.

### Fatty acid composition examination

37 fatty acid compositions were examined in the above 3 transgenic and 1 control fetuses in five tissues, including liver, heart, brain, gluteus and testis. Approximately 0.5 g tissue was grinded using a mortar and pestle with 3 ml chloroform/methanol (v/v = 2/1), the mixture was placed a while until separated layer, transferring aqueous phase into a new tube and keeping it at 4°C. Supplemented 2 ml chloroform/methanol in the residual, shaking for homogeneity, standing over the night, the top organic layer was removed carefully and evaporated under nitrogen. The subside was resuspended in 2 ml n-hexane, adding 400 μl KOH methanol saturated solution, shaking for 2 min, stewing 10 min, then 700 μl supernatant was analyzed by an HP-6890 Series II gas chromatograph with a HP-88 capillary column (0.20 μm × 100.0 m × 0.25mm) attached to an HP-5971 mass spectrometer (GC-MS). Relative compositions of fatty acids contents were calculated according to recorded peaks from a variety of analyzed fatty acids.

### Fetal skin cells were isolated and cells were sorted

Skin cells from ear and haunch was isolated from 4 fetuses through hysterotokotomy on about pregnant day 100 (Numbered as Control, DSH1, DSH2 and DSH3) and 2 newborn infants (Numbered as DSH4 and DSH5), dissociated with 1 mg/ml collagenase Ⅳ and cultured in 100 mm-petri dish in medium DMEM supplemented with 10% inactivated fetal calf serum (Gibco, USA), 100 U/ml of penicillin and 100 U/ml of streptomycin sulfate at 38°C, 5% CO_2_. Cells were harvested when covered the bottom of the dish, using 0.05% Trypsin/EDTA. Cells were filtered through cell strainer caps (35 μm mesh) to obtain a single cell suspension. They were analyzed and sorted by fluorescence-activated cell sorter (BD Sorp Aira III). The green fluorescence was detected using 490 LP and 510/20 filters. All the analyses were repeated in triplicate. Prior to sorting, nozzle, sheath and sample lines were sterilized with 70% ethanol, followed by washes with sterile water to remove remaining ethanol. The sorting cells were cultured in DMEM supplemented with 10% fetal bovine serum, 100 U/ml penicillin, and 100 U/ml streptomycin sulfate. All cells were cultured at 38°C under 5% CO_2_. When 70–80% confluence, cells were harvested and stored at -80°C for DNA, RNA and protein extraction.

### Prediction of CpG island and core area of CAG promoter

We examined CpG islands and CpG sites in CAG promoter by MethPrimer software online (http://www.urogene.org/cgi-bin/methprimer/Methprimer.cgi) with default settings (window: 100; shift: 1; Obx/Exp: 0.6; GC%: 50%). The core region of CAG promoter was analyzed by computational promoter scan prediction tools (http://www-bimas.cit.nih.gov/molbio/proscan/).

### Bisulfite sequencing

About 1 × 10^7^ cells, homogenized in 600 μl DNA extracting buffer (100 mmol/l EDTA, 10 mmol/l TriseHCl, pH 8.0, 2% SDS, 300 mmol/l NaCl), were used to extract DNA with the ammonium acetate method. 5 μl proteinase K (20 mg/ml) was added in each tube, incubating at 55°C for 8 hours until complete digestion, subsequently blending with 200 μl (7.5 mmol/l) ammonium acetate solution, followed by centrifugation at 12,000 rpm for 10 min at 4°C. The supernatant was transferred into another tube containing 600 μl chilled isopropanol, followed by identical centrifugation. Finally the sediment was washed twice with 70% ethanol, the DNA pellet was dried, then dissolved in 100 μl ddH2O and stored at -20°C, prepared for bisulfite modification.

The resulting DNA (1mg) was treated with the EZ DNA Methylation-Gold Kit (D5006, Zymo Research) according to the manufacturer's instruction. The bisulfite treated DNA was amplified by PCR for methylation investigation of cytimidine. The PCR primers were shown in Table 1. The PCR products were separated on 1% agarose gel and purified. Then the products were cloned in pMD-19 T vector (TaKaRa, Japan), transformed and spread plates, and the positive clones were obtained through colony PCR screening. At least twelve successful sequencings were obtained and aligned by Vector NTI Suite 11.0 (Invitrogen). Methylation status of every CpG site was counted and percentage of methylation was calculated.

**Table 1:** Primer information for scanning methylation status in CAG promoter region.

Primer name	Sequence (5’→3’)	Amplicon position (nt)
		EGFP negative (-) or positive cells
CF175 (forward)	GGTAAATGGTTCGTTTGGTTGA	78–195
CR176 (reverse)	ATAATCCACCCATTAACGTCAATAA	
FF1177 (forward)	GTTAATAGGGATTTTTTATTGA	115–320
FR1179 (reverse)	AAAAAATAAAAAAAATAAAACAAAAC	
CF213 (forward)	TTTTTAATTATTTTGTGTAGCGATG	445–742
CR214 (reverse)	CCGCTCACCTATAAAAATAACG	
CF179 (forward)	TTATAGGTGAGCGGGCGG	729–973
CR180 (reverse)	CGAAACGCACAAAACCCC	(-)
CF195 (forward)	TATAGGTGAGTGGGTGGGATG	730–976
CR196 (reverse)	ACACAAAACACACAAAACCCC	(+)
CF30 (forward)	CGGGGTTTTGTGCGTTTC	955–1153
CR31 (reverse)	AACCGAACCGTACTCAACAACTC	(-)
CF112 (forward)	TGGGGTTTTGTGTGTTTTGTGT	955–1156
CR113 (reverse)	CAAAACCAAACCATACTCAACAACT	(+)
CF49 (forward)	GAGTTGTTGAGTACGGTTCGGTT	1131–1368
CR50 (reverse)	AAATCCCTACGCCCTCTCG	
CF51 (forward)	GCGAGAGGGCGTAGGGAT	1349–1602
CR52 (reverse)	GCCGATCACACGCCAAAA	(-)
CF79 (forward)	GTGTGAGAGGGTGTAGGGATTT	1347–1602
CR80 (reverse)	ACCAATCACACACCAAAAACC	(+)

### Western blot

For western blot analysis, total protein was isolated from sorted cells. Cells were collected from 6-well plate, samples were boiled in 0.0625 M Tris hydrochloric acid (pH 6.8), 2% sodium dodecyl sulfate, 0.001% bromophenol blue, and 5% 2-mercaptoethanol for 10 min. Before western blot, all the proteins were quantified by the BCA (Bicinchoninic acid) method. IRES maintained independent expression of fat-1 and EGFP, without commercial fat-1 protein antibodies, anti-EGFP monoclonal antibody (ComWin, China) was selected as the primary antibody for the western blot experiments. The β-actin antibody (Santa, USA) was used as the loading control. The proteins were separated by polyacrylamide gel for 50 min at 100 V followed 1.5 h at 120 V respectively, and then electrophoretically transferred onto a nitrocellulose membrane. After blocking with 1% bull serum albumin, the membrane was incubated with the primary antibody and then subsequently with a horse radish peroxidase goat anti-mouse lgG antibody (Abgent, USA) for 2 h at room temperature for each antibody. Then, the signals were visualized with chemiluminescence using imaging systems (ChemiDoc XRS1) (Bio-Rad, USA).

### Real-time quantitative RT-PCR

The tissues (100 mg each sample) including liver, spleen, kidney, heart, lung, brain, testis, crureus, gluteus and dorsal muscle were collected from above 5 transgenic and 1 control animals. The samples were homogenized in TRIZOL reagent (Invitrogen, USA), and total RNA was isolated according to the manufacturer’s instruction. Total RNA was incubated with RNase-free DNase I (Roche, Switzerland) to eliminate contaminated genomic DNA before being reverse transcribed into cDNA using random hexamer primers and M-MLV Reverse Transcriptase (Promega, USA).The real-time fluorescence quantitative reverse transcription polymerase chain reaction method (qRT-PCR) was established in a CFX96 Real-Time PCR Detection System (Bio-Rad, USA). All the qRT-PCR experiments were in triplicate. β-actin was used as an internal control. The primers were shown in Table 2. The program and system of qRT-PCR were described previously [19].

**Table 2:** Primer sequences and their designated application in this study.

Primer name	Sequence (5’→3’)	Amplicon length (nt) and primer information
*β-actin*		
SFA1498 (forward)	GCAGATGTGGATCAGCAAGC	145
SRA1499 (reverse)	AGGCCAATCTCATCTCGTTTT	qRT-PCR
*fat-1*		
FF1338 (forward)	CTGAAGTGGTTCCCCGTGTA	95
FR1339 (reverse)	ACTCTTTCGCTGTTCCGCA	qRT-PCR
FF1276 (forward)	CGTCAACGCCAACACC	946
FR1273 (reverse)	GGGCTCCAGCACTTTC	Confirming sequence
*ME1*		
F1727 (forward)	ATCCCGTTACCCTTCCGAGT	91
R1728 (reverse)	GCCACGACACCAAGAGCAA	qRT-PCR
*SCD-1*		
F1729 (forward)	GCCGTGGTATCTATGGGGTG	150
R1730 (reverse)	CGGGGGTTGATGGTCTTGT	qRT-PCR
*ACBP*		
F1731 (forward)	AAGACCAAGCCAGCAGATGAG	78
R1732 (reverse)	TGTATTTATGTCACCCACAGTTGC	qRT-PCR
*FABP1*		
F1785 (forward)	GTCCAGACCCAGGAGAACTACG	97
R1786 (reverse)	TTTCCGACACCCCCTTGAC	qRT-PCR
*FABP3*		
F1735 (forward)	ATTCACTCGGTGTCGGTTTTG	170
R1736 (reverse)	GTCATCTGCCGTGGTCTCATC	qRT-PCR
*LPL*		
F1735 (forward)	TGAAGACTCGTTCTCAGATGCC	149
R1736 (reverse)	GGTAAAAGGGATGTTCTCGCTC	qRT-PCR
*ACS*		
F1739 (forward)	CTTGGAGGAGCCGAGAGAGTT	158
R1740 (reverse)	CGTTGTAGCAGATGTTGGTAGTTG	qRT-PCR
*FATP1*		
F1741 (forward)	TGAGGTGGAAGGCGTGCTA	92
R1742 (reverse)	TGCCTGCTTTGCCCTCTACT	qRT-PCR
*FATP4*		
F1779 (forward)	TTTGAGGGTACAGACACGCACT	148
R1780 (reverse)	GCCACAGACCCACAAACTCAT	qRT-PCR
*CD36*		
F1745 (forward)	ACACAGTTTCTTTCCTACGACCC	131
R1746 (reverse)	AAATGTGCTTGGATAGAGATGGG	qRT-PCR
*OLR1*		
F1781 (forward)	GTCCTTTGTCTGGGATTACTGGT	156
R1882 (reverse)	GGAAGATTTTTCTGATTGGCG	qRT-PCR
*Bien*		
F1749 (forward)	ATCCGTCTACGAAACCCGC	119
R1750 (reverse)	GCTCCACAAATCACAATGGCT	qRT-PCR
*Thiolase B*		
F1751 (forward)	GTGGTAGTGGATGGTGTTCGC	98
R1752 (reverse)	AAACCCGAAAGTGCTGCTCTA	qRT-PCR
*SCP2*		
F1753 (forward)	TTTTGTCAAGCCCAGATAGCG	81
R1787 (reverse)	CATCAAAGCCAGTAAGTCCGAGT	qRT-PCR
*ACOX1*		
F1755 (forward)	GCAGAAAAAGCAAGGAGGTAGC	168
R1756 (reverse)	AAGAATACAAGAGACACAAACGCC	qRT-PCR
*CPT-1B*		
F1757 (forward)	TGTGGCAGATGATGGCTACG	157
R1758 (reverse)	TTGGAAAAGATTGGCGATGTC	qRT-PCR
*CPT-2*		
F1759 (forward)	CGACATTTGTTTGCTCTGCG	178
R1760 (reverse)	CAATGCCCAAGCCATCAGA	qRT-PCR
*LCAD*		
F1761 (forward)	GCCCAGGTTTTAGTCTTCATTCA	137
R1762 (reverse)	CCCAGGCTCTGTCATTGCTAT	qRT-PCR
*MCAD*		
F1763 (forward)	CACCGAAATACCTATTATGCCTCT	98
R1764 (reverse)	CCATTGCCTCCAAAAATCTGA	qRT-PCR
*Perilipin1*		
F1883 (forward)	ACGAGGAGCACACGGACACT	173
R1784 (reverse)	CCACATCACGACTGAGACGG	qRT-PCR
*ADIPO*		
F1767 (forward)	TGCTCTTCACCCACGACCA	139
R1768 (reverse)	TTATCCGCATAGACCCCATTG	qRT-PCR
*MMP-1*		
F1769 (forward)	GCTGCTTATGAGGTTGCCG	143
R1770 (reverse)	ATGCTCTTCACCGTTCTTGGA	qRT-PCR
*DNMT 1*		
CF397 (forward)	GAGGAGGCTGCCAAGGACT	134
CR398 (reverse)	CAAACACCGCATACGACACAC	qRT-PCR
*DNMT 3A*		
CF441 (forward)	CTTGGAGAAGCGGAGTGAGC	138
CR442 (reverse)	GTGCAGCAGCCATTCTCTACAG	qRT-PCR
*DNMT 3B*		
CF401 (forward)	AGCCCCTACCTCACCATCG	156
CR402 (reverse)	CTGATACTCGGTGCTGTCTGC	qRT-PCR
*DNMT 3L*		
CF403 (forward)	CCCAGACATTTGTTCAGACGGT	122
CR404 (reverse)	CCACAACGCCCACCTACCT	qRT-PCR
*EGFP*		
CF459 (forward)	AGTGCTTCAGCCGCTACCC	168
CR460 (reverse)	GCTCGATGCGGTTCACCAG	qRT-PCR

### Statistical analysis

Every relatively independent experiment was repeated at least three times, and the results were represented as mean ± standard average (SE). Student’s *t*-test was used to test differences in methylation levels in CAG promoter region and mRNA expressions of relevant genes. The significant difference was considered at P < 0.05, and the extremely significant level was P < 0.01.

## Results

### Characterizations of fat-1 transgenic cell lines and embryo transfer

**Fig 1:**
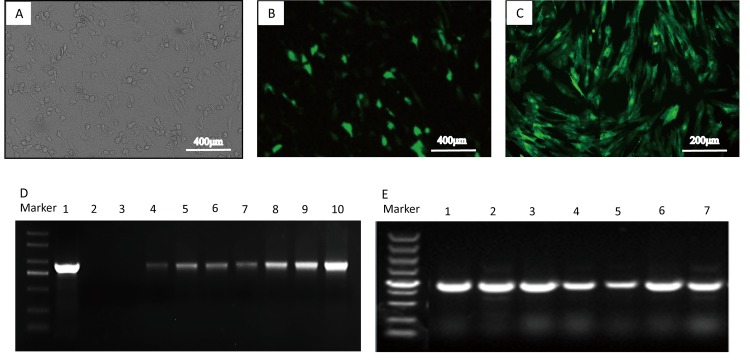
Identification of pCAG-fat-1-IRES-EGFP transfected cells. Sheep fetal fibroblasts were transfected with pfat-1 expression vector and screened with G418. **(A)** Phase-contrast image of transfected cells. **(B)** Fluorescent image of transfected cells. **(C)** Cell line obtained by cell screening. **(D)** fat-1 gene detection in genomic DNA by PCR. Plasmid pfat-1 as positive control in lane 1, ddH_2_O as blank in lane 2, non-transgenic cells as negative control in lane 3, and lane 4–10 are from 7 EGFP-positive cell lines. **(E)** Investigation of fat-1 gene mRNA expression by RT-PCR. cDNAs are from 7 EGFP-positive cell lines.

After transfection and selection, seven pfat-1 transfected positive monoclonal cell lines were obtained (**Fig 1**). According to PCR examination results, fat-1 gene was integrated into sheep genome and expressed in all of the cell lines. One transgenic cell line was employed as donor cells, producing transgenic livestock by nuclear-transfer. Totally, 441 transgenic cloned embryos at the 1–2 cell stage were transferred to the oviducts of 52 synchronism recipient sheep.

### Generation and examination of fat-1 transgenic sheep

**Fig 2:**
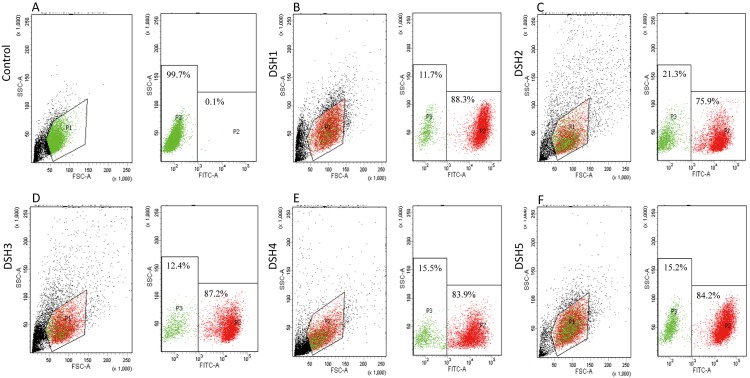
Investigation of EGFP-positive profiles in transgenic animals by flow cytometry. The cells from the leg skin tissues of somatic nuclear transfer fetuses were isolated and cultivated. The percentage of fluorescent cells was examined by flow cytometry. **(A)** Non-transgenic skin cells served as control. **(B-F)** The positive situation in leg skin cells of transgenic animals in DSH1, DSH2, DSH3, DSH4 and DSH5, respectively. The black spots indicate total cells. The red show EGFP-positive cells, and the green represent EGFP-negative cells.

On day 70 after embryo transfer, 10 (19.2%) recipient sheep were pregnant by B-sonography detection. From gestation to birth, two group samples were collected. One group were gathered by caesarean on day 100 after gestation, named as DSH1, DSH2 and DSH3. Another group were natural birth, named as DSH4 and DSH5. The cells from the skin tissues of DSH1, DSH2, DSH3, DSH4 and DSH5 were separated and cultivated. The percentage of fluorescent cells in the leg skin cells was detected by flow cytometry, and there were rather different in various individuals (**Fig 2**), DSH1 (88.3%), DSH2 (75.9%), DSH3 (87.2%), DSH4 (83.9%) and DSH5 (84.2%). Even in various location of the same tissue, there was different. The EGFP positive cells in the skin from the ear was 80.4%, but it only was 75.9% in that from leg (**S1 Fig**). Cell nuclei of those cloned embryos were from single cell clones, however, the expression patterns of exogenous gene in nuclear transplantation embryos were different in various individuals and tissues.

### fat-1 gene expression in various tissues and different developmental stages of transgenic sheep

**Fig 3:**
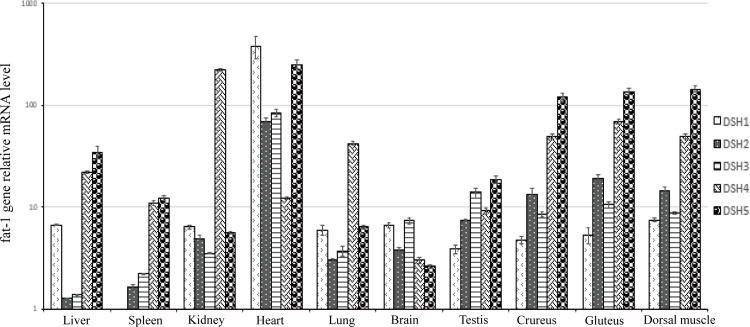
fat-1 gene expressions in different individuals, disparate tissues, various development stages. mRNA expressions of fat-1 gene in transgenic sheep embryos were investigated by qRT-PCR. mRNA expression was relative to that in DSH1 spleen. fat-1 gene expression in liver, spleen, kidney, heart, lung, brain, testis, crureus, gluteus and dorsal muscle tissues in 100-day fetuses (DSH1, DSH2 and DSH3) and neonates (DSH4, DSH5).

The expression quantity of fat-1 mRNA in 10 tissues was very different in single individual, but it was the similar trend among individuals at the same developmental stage (**Fig 3**). mRNA expression patterns is different between day 100 fetuses and neonatal lambs. With the constant development, mRNA expressions of fat-1 gene obviously increased in liver, spleen, kidney, lung, crureus, gluteus and dorsal muscle, and clearly reduced in brain (**S2 Fig**). The results indicated that the differences existed in the inter-individual as well as inter-tissue from the identical genetic background.

### Omega-3 fatty acid content increases in fat-1 transgenic lambs

**Fig 4:**
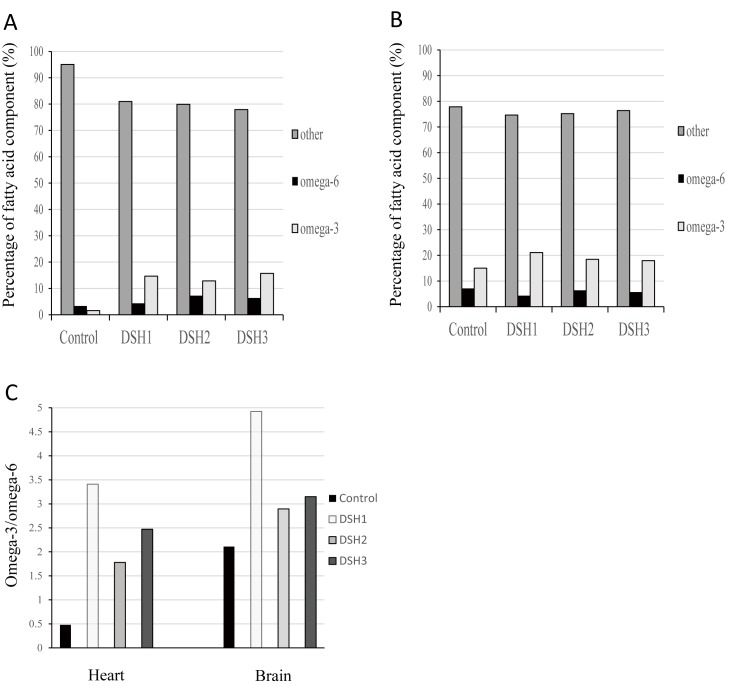
The omega-3 and omega-6 fatty acid content patterns in two tissues. **(A)** The percentages of omega-3, omega-6 and other fatty acids in liver from DSH1, DSH2, DSH3 and control individuals. **(B)** The percentages of omega-3, omega-6 and other fatty acids in brain. **(C)** Omega-3/omega-6 ratio. fat-1 gene increases the ratio of omega-3/omega-6 fatty acids.

The major function of fat-1 gene is catalyzing omega-6 fatty acids into omega-3 fatty acids. Here, we investigated the association between the expression of fat-1 gene and the content of 37 fatty acids in liver, heart, brain, gluteus and testis tissues. The results indicated that the content of omega-3 fatty acids obviously increases (**Fig 4 and S3 Fig**), which meant that fat-1 transgenic sheep is successful and exogenous gene exerts proper function. Further analysis showed that the ratio of omega-3 and omega-6 fatty acids is positive correlation with fat-1 expression (**S3E Fig**).

### Eighteen genes of PPAR pathway expression analysis in five tissues

As ligand, unsaturated fatty acids play an important role in the PPAR pathway. In this study, we further explored the influence of fatty acid composition change on PPAR pathway. mRNA expression patterns of eighteen genes in PPAR pathway were investigated in five tissues in six animals by qRT-PCR. The genes can be divided into four categories: lipogenesis (ME1, SCD1), fatty acid transport (ACBP, FABP13, LPL, ACS, FATP1, CD36 and DLR1), fatty acid oxidation (Bien, Thiolase B, ACOX, CTP-2, LCAD and MCAD) and adipocyte differentiation (ADIPO, MMP-1) (**S4 Fig**). Although gene expressions are obvious differences in inter-tissue and inter-gene, the results indicated fat-1 largely affects fatty acid metabolism network.

### Cell sorting and expression examination

**Fig 5:**
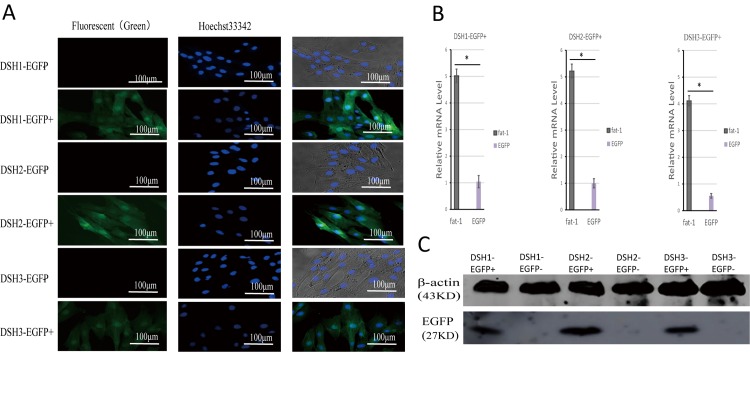
EGFP-positive and -negative cells were sorted by flow cytometer from skin cells of 3 fetuses and expression characteristics were investigated. **(A)** The green indicate EGFP-positive cells, the blue show cell nucleuses. Scale bars = 100 μm. **(B)** mRNA expression levels of fat-1 and EGFP genes in EGFP-positive cells. The results are presented as the mean± standard deviation (n = 3). Statistical difference (P≤0.05) is indicated by asterisks (*). **(C)** Western blot analysis of EGFP protein expression in EGFP-positive and -negative cells.

EGFP positive and negative cells were sorted by flow cytometer from DSH1, DSH2 and DSH3 skin cells, and the sorted cells were cultured and proliferated *in vitro*. The green fluorescence could been seen in all the EGFP positive cells under fluorescence microscope, but not in the EGFP negative ones (**Fig 5A**). mRNA expressions of fat-1 and EGFP genes were not detected in EGFP negative cells. The expression level of fat-1 gene was higher than that of EGFP gene in the corresponding positive cells, with the same tendency in the three individuals, so EGFP can be a marker to explore the heredity in transgenic animals (**Fig 5B**). Western blot analysis confirmed EGFP protein expression in EGFP positive cells not in EGFP negative ones (**Fig 5C**).

### Hypermethylation of CAG promoter region 721–1346 nt prevents exogenous gene expression

**Fig 6:**
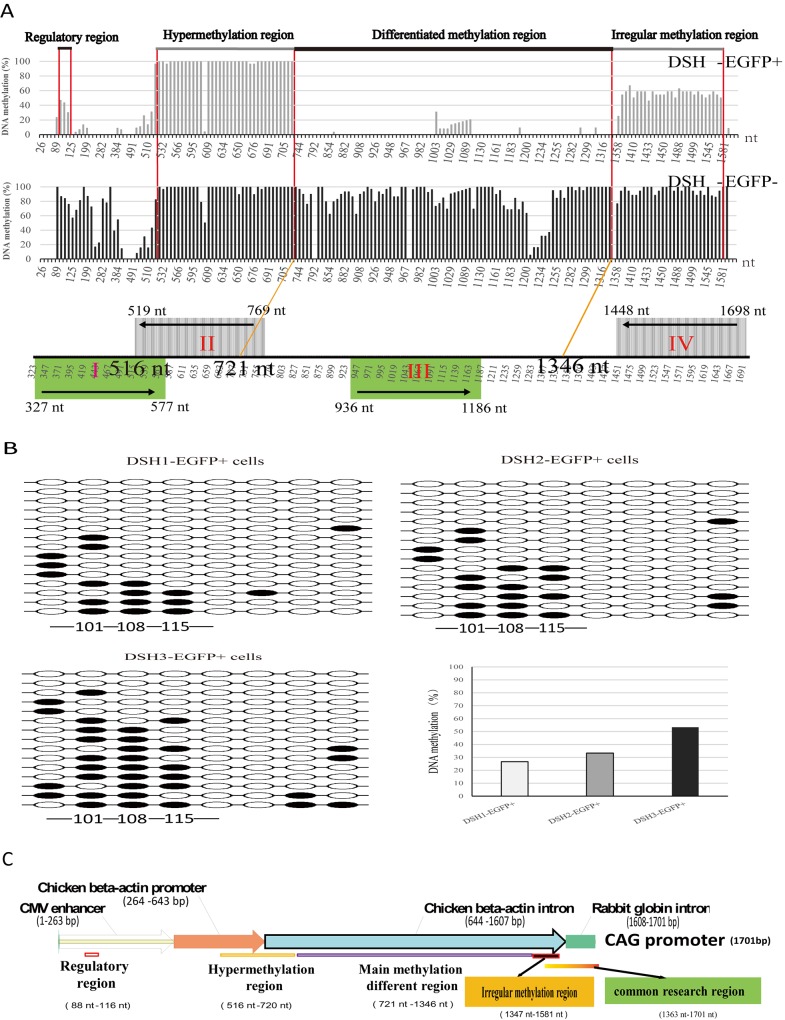
Fine-mapping of DNA methylation in the whole CAG promoter by bisulfite sequencing. **(A)** DNA methylation status in the whole CAG promoter region in EGFP-positive and negative skin cells from 3 100-day fetuses. The full-length region was scanned by 7 pairs of primers. All the PCR products were ligated into T-vector. At least 12 successful sequencings were obtained for data analysis. DNA methylation rate represents ratio of methylated counts in successful sequencings in corresponding site. DNA methylation (%) shown in the upper and middle figures is the average of three samples. Horizontal axis indicates the 176 CpG sites. The bottom figure shows core promoter regions predicted by online software and alignment with methylation distribution. **(B)** Methylation levels at CpG site 101, 108, and 115. Each sample carried out 15 successful sequencing. Hollow and solid circles stand for unmethylated and methylated cytosines, respectively. The histogram exhibits the percentage of methylation at the 3 selected CpG sites. **(C)** Schematic diagram of structural regions and DNA methylation status of CAG promoter. The CAG promoter is made up of four regions: CMV enhancer (1–263 nt), chicken β-actin promoter (264–643 nt), chicken β-actin intron (644–1607 nt) and rabbit globin intron (1607–1701 nt). The CAG promoter methylation patterns include: regulatory region (108–116 nt, 3 CpG dinucleotides), hypermethylation region (516–720 nt, 37 CpG dinucleotides), main methylation different region (721–1346 bp, 85 CpG dinucleotides), common research region (1374–1600 nt, 30 CpG dinucleotides) and irregular complex methylation region (1347–1664 nt, 33 CpG dinucleotides).

To explore regulatory mechanisms of fat-1 gene expression, we thoroughly investigated the methylation status of the whole CAG promoter in EGFP positive and negative cells by bisulfite sequencing method (**Fig 6A upper and middle**). On the basis of correlation analysis of the methylation status of CAG promoter and the expression level of EGFP, we found that the methylation levels of three CpG loci (101, 108 and 115 nt) are negative correlation with exogenous gene expression quantity (**Figs 6B and 5B**). Interestingly, DNA sequence (466–526 nt) forms hairpin structure in front of the hypermethylation region (512–720 nt). Highest methylation levels (512–720 nt, encompassing 37 CpG dinucleotides) were observed not only in positive cells but also in negative cells (**Fig 6A upper and middle**). Most importantly, hypermethylation was observed in EGFP negative cells and hypomethylation in EGFP positive ones from 721–1346 nt, including 85 CpG dinucleotides (**Fig 6A upper and middle**). These findings suggested that exogenous gene expression was determined by the methylation status from 721–1346 nt and modulated by methylation levels at 101, 108 and 115 nt sites in CAG promoter. To compare with theoretic analyses, CpG islands and core promoter regions were predicted by online programs. CAG promoter contains two CpG islands and 176 CpG dinucleotides. The first island was located from 101 nt– 375 nt which contained the regulatory methylation sites (101, 108 and 115 nt), and the second one from 386 nt– 1593 nt which contained the determinant differential methylation region (721–1346 nt). Four core promoter regions were predicted, just one (936–1189 nt) was located in the differential methylation region (**Fig 6A bottom**). To overall understand functional structure of CAG promoter, we drafted the composition and methylation status in CAG promoter (**Fig 6C**). These researches accomplish fine-mapping of the whole CAG promoter methylation, which provide markers for transgenic animals with CAG promoter and offer strategies for other gene expression regulation studies.

### DNMT 1 maintains high methylation and prevents exogenous gene expression in EGFP negative cells

**Fig 7:**
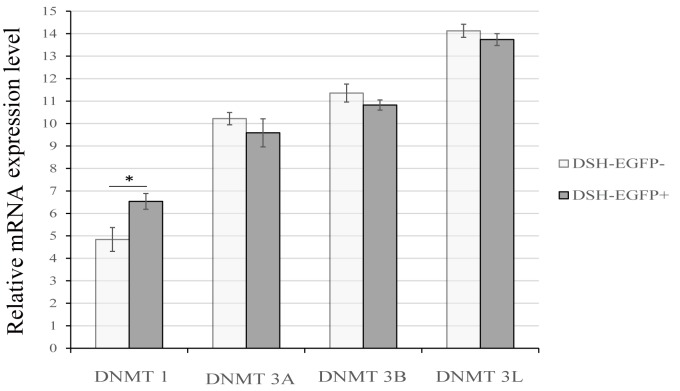
mRNA expressions of 5 DNA methyltransferases in EGPP-positive and negative cells. Samples are obtained by flow cytometer from 3 100-day fetuses. qRT-PCR was employed to quantify mRNA expressions. The data were shown by ΔCTs of target genes minus those of β-actin in corresponding samples. The results are presented as the mean± standard deviation (n = 3). Statistical difference (P≤0.05) is indicated by asterisks (*).

DNMTs are involved in the epigenetic control of DNA methylation process, major catalysts of DNA methylation. mRNA expressions of four DNMTs were examined by qRT-PCR in EGFP positive and negative cells. mRNA expression of DNMT 1 in negative cells was significantly higher (3.25-folds) than that in positive ones (P ≤ 0.05) (**Fig 7**). DNMT 1 is the most abundant DNA methyltransferase in mammalian cells, and considered to be the key maintenance methyltransferase in mammals. It predominantly methylates hemimethylated CpG dinucleotides in the mammalian genome. DNMT 3B transcripts in negative cells were obviously lower (0.46-fold) than those in positive ones (P ≥ 0.05). DNMT 3B can mediate methylation-independent gene repression. DNMT 3B can also interact with DNMT 1, which might be a co-operative event during DNA methylation. The results demonstrated that hypermethylation of CAG promoter is mainly maintained by DNMT 1 in EGFP negative cells, which prevent mRNA expression of exogenous gene.

### EGFP positive and negative cells belong to different types

**Fig 8:**
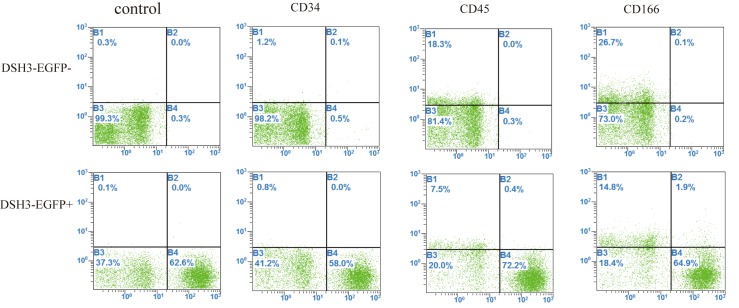
Cell type was analyzed through cell surface antigen markers CD34, CD45 and CD166 by flow cytometry. Marker CD34 was barely detectable. Marker CD45 and CD166 occupied 7.9% and 16.7% in EGPF-positive cells, 18.3% and 26.8% in EGFP-negative ones, respectively. The experiment was carried out triplicately. The positive cells of surface antigen share the B1 and B2 areas.

We detected the cell surface antigens CD34, CD45 and CD166 by means of cytometry technology in EGFP positive and negative cells. CD34 marker was very low in two type of cells. The expression percentages of CD45 and CD166 in EGFP negative cells were higher than those in EGFP positive cells, which suggested they belong to different cell types (**Fig 8**). CD45 is a receptor-linked protein tyrosine phosphatase that is expressed on all leucocytes. CD166 has been used as a potential cancer stem cell marker, so EGFP negative cells might be rather primitive cells.

## Discussion

fat-1 gene serves as a perfect target gene in transgenic mammals due to its excellent economic value. In the present study, pfat-1 vector was employed for generation of fat-1 transgenic sheep. In pfat-1, artificial promoter CAG simultaneously drives mRNA expressions of fat-1 and EGFP linked by IRES sequence [20]. IRES-dependent second gene expression is significantly lower than cap-dependent first gene expression in a bicistronic vector [21]. In **Fig 5B**, mRNA expression of fat-1 gene is over ten times higher than that of EGFP gene in fetal skin cells, which indicated that the vector works very well in the present study and EGFP can be a very good indicator of exogenous target gene expression.

To further verify the features of fat-1 transgenic sheep, we examined mRNA expression and function of fat-1 gene. The results showed the positive correlation between mRNA expression quantity and omega-3 fatty acid content (**Figs 3 and 4**). Individuals obtained by somatic nuclear transfer develop various types and differentiation of cells. Different expressions of fat-1 gene in various tissues implied that fat-1 expression displays cell otherness. fat-1 expression increases during embryonic development, which indicated that fat-1 expression rises in the higher differentiated cells.

Why does exogenous genes express or silence in different cells in same tissue? To address the issue, cell sorting were performed by flow cytometry, almost 80% leg skin cells of transgenic sheep are EGFP positive cells, and few cells (20% or so) are EGFP negative (**Fig 2**). Few negative cells in positive transgenic animals are also observed in other transgenic animal tissues [22–24]. The developmental program of embryogenesis is controlled by both genetic and epigenetic mechanisms. The regulation of higher-order chromatin structures by DNA methylation and histone modification is crucial for genome reprogramming during early embryogenesis and gametogenesis [25–27]. As a stable repressive mark, DNA methylation, catalyzed by the DNMTs, is regarded as a key player in epigenetic silencing of transcription. DNA methylation may coordinately regulate the chromatin status via the interaction of DNMTs with other modifications [28]. In the embryonic development in human and mouse, hypermethylation appears in sperm and ovum stage, following hypomethylation from 2 cell to blastula, and *de novo* methylation [29, 30]. In the embryonic development in somatic nuclear transfer animals, methylation experiences similar process [31]. To explore how promoter methylation controls the exogenous gene expression in details, methylation statuses of full-length CAG promoter regions in two types (EGFP positive and negative) of cells were thoroughly investigated by bisulphate sequencing method. The results disclosed that exogenous gene expression was determined by the methylation status from 721–1346 nt (85 CpG loci) and modulated by methylation levels at 101, 108 and 115 nt sites in CAG promoter. In the previous primary studies on correlation between CAG promoter methylation status and EGFP expression in sheep and mouse [18, 32], both of them focused on 1374–1600 nt region (30 CpG loci). Compared with our fine-mapping, this region seems to negative correlation between average methylation status and gene expression level (**Fig 6**), however, methylation status is actually irregular in this region in the EGFP positive cells (**S5 and S6 Figs**). Furthermore, most researchers investigated methylation status with DNA derived from tissues, in fact, more than two types of methylation profiles exist in tissues in consistent with cell types.

Why does the promoter region methylation status of exogenous gene generate differentiation in different cells (EGFP positive and negative)? It is reported that *de novo* methylation correlate DNMTs in cell division, DNMT1 can independently maintain methylation of most CpG sites [33] in the DNA replication [34, 35]. In the present study, DNMT 1 expression is significantly higher (3.25-folds) in EGFP negative cells than that in positive ones (P ≤ 0.05), which ensures hypermethylation and prevents exogenous gene expression in negative cells. We also found that DNMT 3B expression is lower (0.46-fold) in negative cells than that in positive ones (P ≥ 0.05). Normally, DNMT 1 and DNMT 3B cooperate to regulate the methylation status in genes, but DNMT 1 exerts dominant role in methylation maintenance [33, 36].

DNMTs expressions might be associated with cell types. We discovered that CD45 and CD166 expressions are higher in negative cells, which belong to cell surface antigen of stem cells [37, 38]. The results indicated EGFP negative cells are more primitive cells. DNA methylation provides a potential epigenetic mechanism for the cellular memory needed to preserve the somatic progenitor state through repeated cell divisions. DNMT 1 maintains DNA methylation patterns after cellular replication and is essential for epidermal progenitor cell function [39].

In summary, exogenous gene expression in transgenic animal depends on cell types and differentiated level. DNMT 1 maintains hypermethylation of CAG promoter specific region (721–1346 nt) and consequently prevents expression of exogenous gene in fat-1 transgenic sheep. The results clarify regulatory mechanisms of exogenous gene expression in fat-1 transgenic sheep, which will be helpful to understand gene expression regulation in transgenic animals.

## Supporting information

S1 Fig Investigating EGFP-positive profile in DSH1 by flow cytometry. (A) Non-transgenic skin cells as control. (B and C) The EGFP-positive patterns were detected in ear and leg skin cells, respectively.
(TIF)

S2 Fig mRNA expression of fat-1 gene in different tissues and developments. (TIF)

S3 Fig The omega-3 and omega-6 fatty acid content patterns in three tissues. (A) Liver. (B) Gluteus. (C) Testis. (D) Omega-3/omega-6 ratio. fat-1 gene increases the ratio of omega-3/omega-6 fatty acids. (E) The relationship of Omega-3/omega-6 ratio and fat-1 mRNA expression level.
(TIF)

S4 Fig The expression patterns of 18 genes which belong to PPAR pathway in different tissues and individuals. (A) The expression patterns of 18 genes in liver tissue from DSH1, DSH2 and DSH3. Four function categories were boxed and under gene names. mRNA expression of OLR1 gene was significantly different from others. (B-E) shows the expression patterns of 18 genes in heart, brain, gluteus and testis tissues from DSH1, DSH2 and DSH3, respectively, most of the gene expression patterns were similarity in the same tissues, differential genes were indicated by hollow triangle. (F-H) displays the expression profiles of 18 genes from DSH1, DSH2 and DSH3 in liver, heart, brain, gluteus and testis tissues, there were significantly different in five tissues from DSH1 and DSH3. However, there were similar in other four tissues in DSH2 except in liver tissue.
(TIF)

S5 Fig Methylation status of EGFP negative cells in CAG promoter region. (A) DSH1. (B) DSH2. (C) DSH3.
(TIF)

S6 Fig Methylation status of EGFP positive cells in CAG promoter region. (A) DSH1. (B) DSH2. (C) DSH3.
(TIF)
