# Supplementary figures and images for: DNMT 1 maintains hypermethylation of CAG promoter specific region and prevents expression of exogenous gene in fat-1 transgenic sheep
Source: PLoS One. 2017 Feb 3;12(2):e0171442. doi: 10.1371/journal.pone.0171442 (PMC5291418; doi:10.1371/journal.pone.0171442)

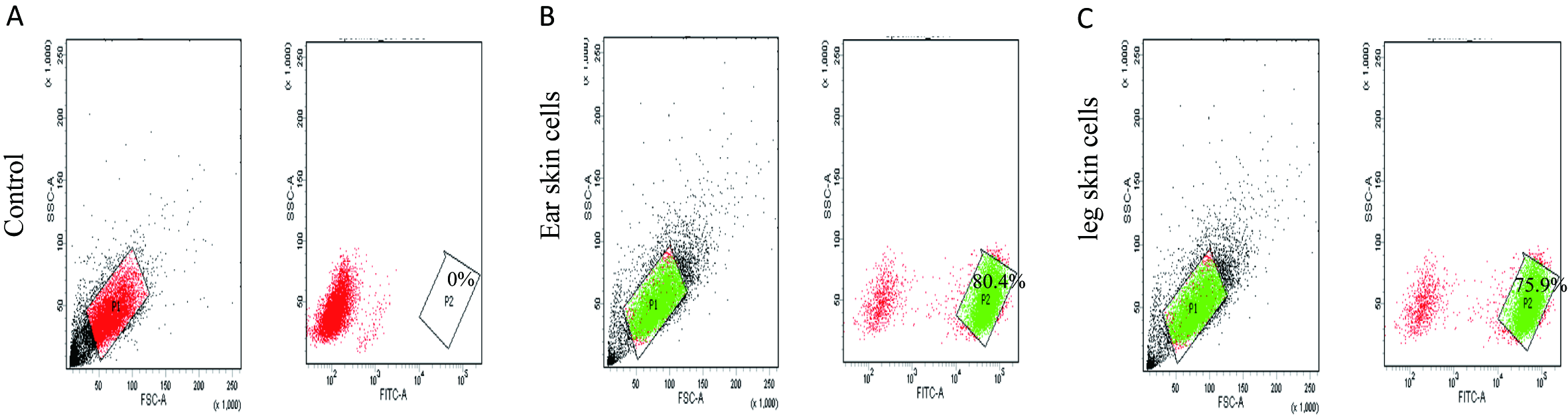

Supplement: S1 Fig — (A) Non-transgenic skin cells as control. (B and C) The EGFP-positive patterns were detected in ear and leg skin cells, respectively. (TIF) [file pone.0171442.s001.tif]

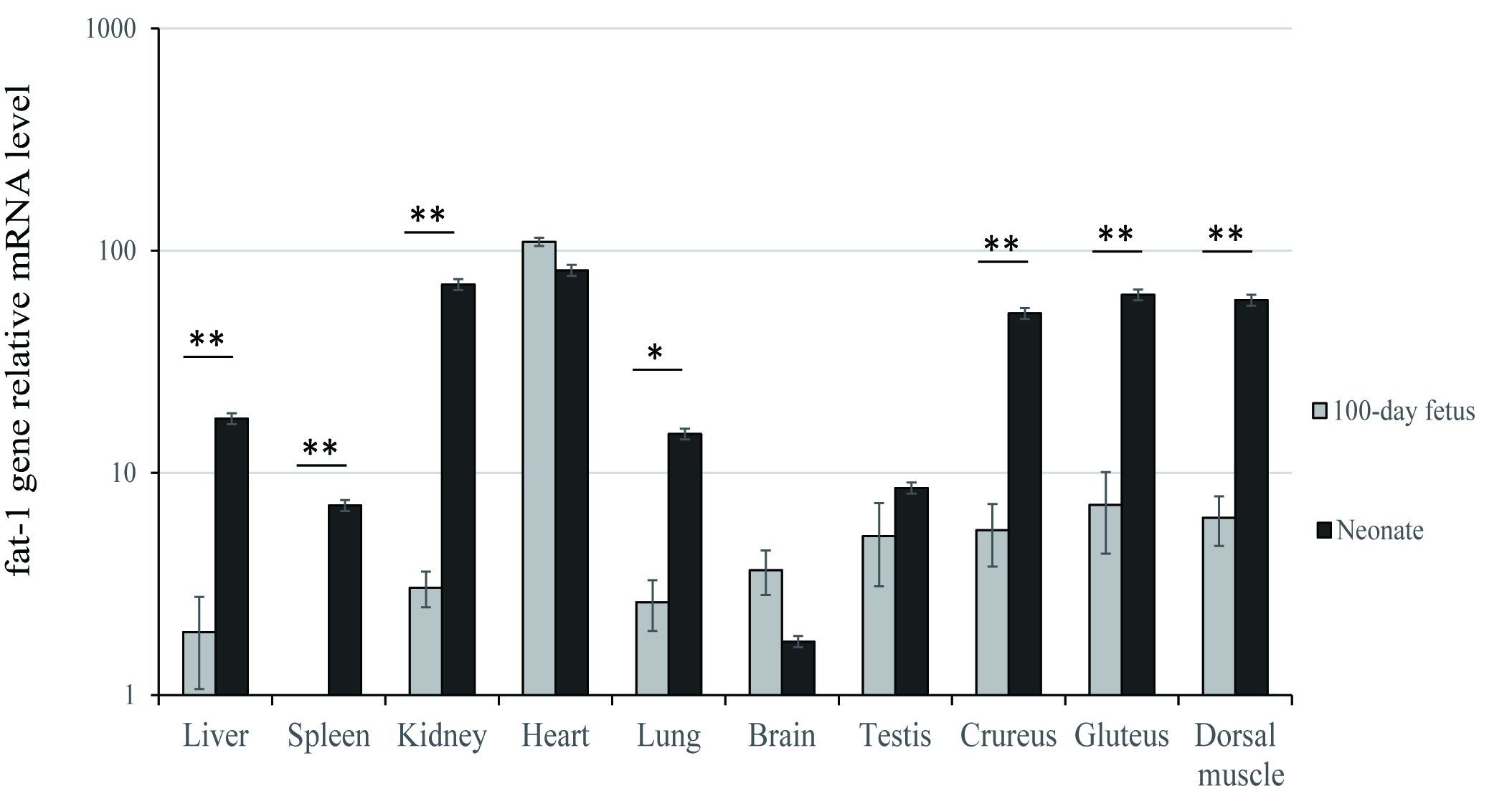

Supplement: S2 Fig — (TIF) [file pone.0171442.s002.tif]

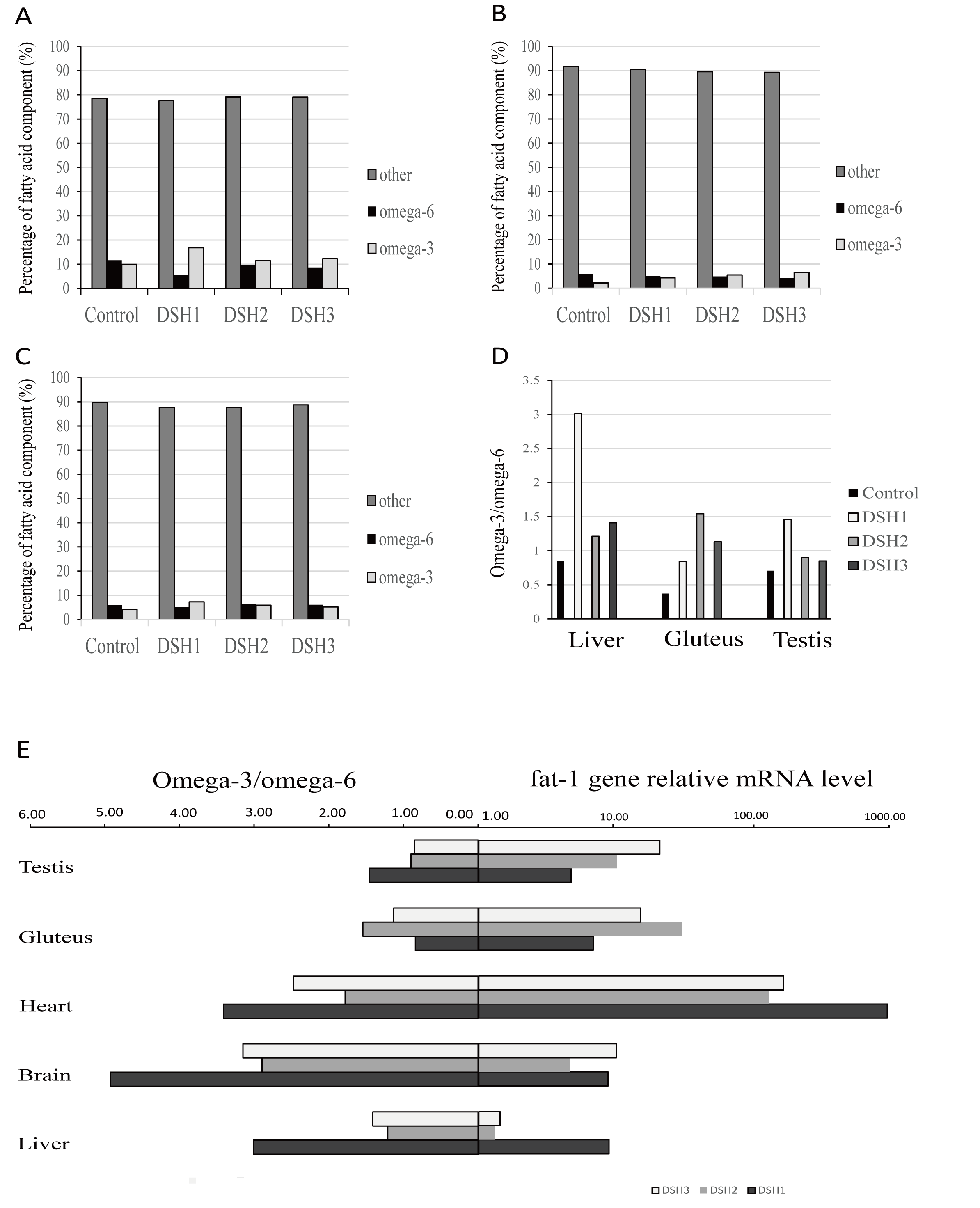

Supplement: S3 Fig — (A) Liver. (B) Gluteus. (C) Testis. (D) Omega-3/omega-6 ratio. fat-1 gene increases the ratio of omega-3/omega-6 fatty acids. (E) The relationship of Omega-3/omega-6 ratio and fat-1 mRNA expression level. (TIF) [file pone.0171442.s003.tif]

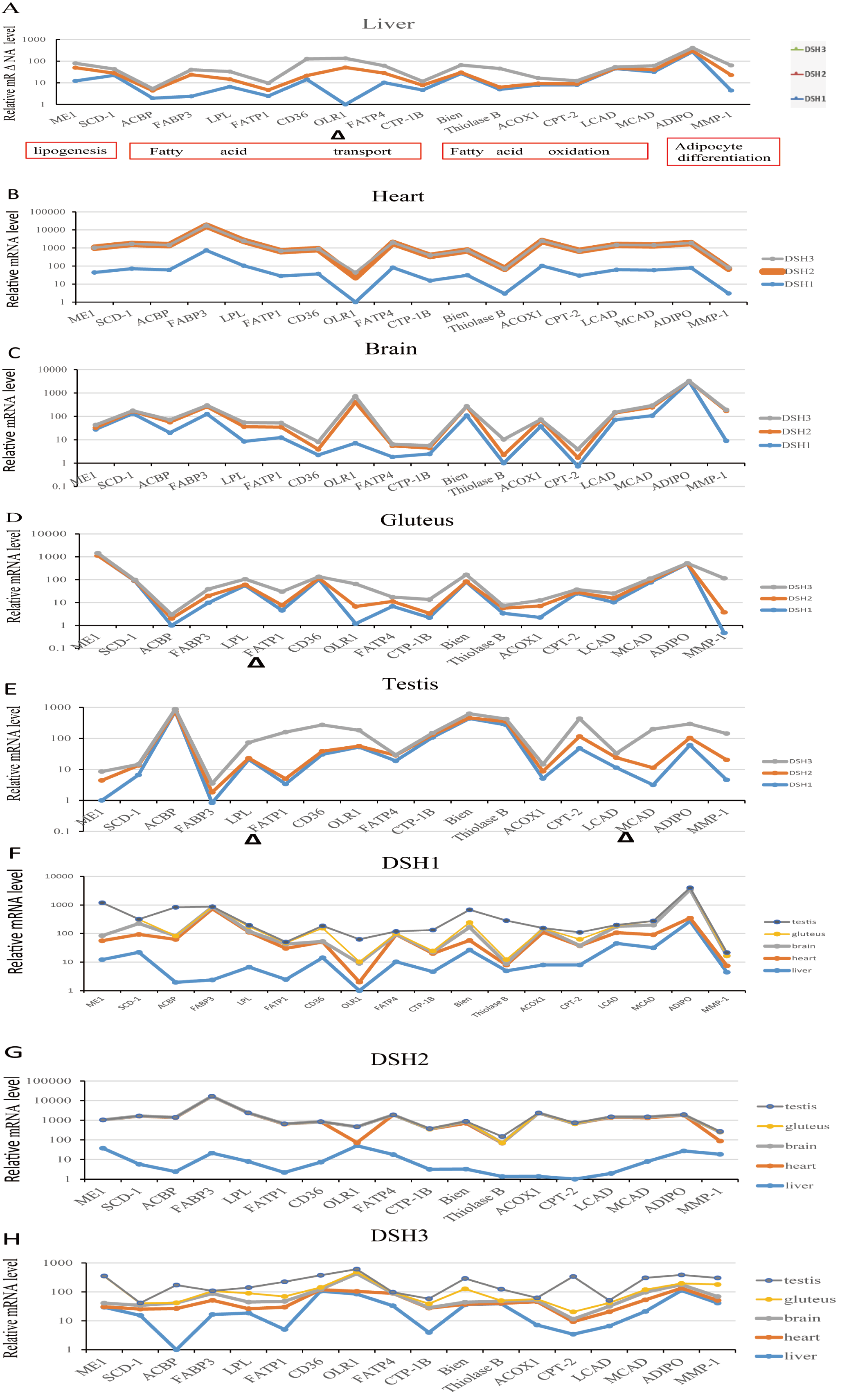

Supplement: S4 Fig — (A) The expression patterns of 18 genes in liver tissue from DSH1, DSH2 and DSH3. Four function categories were boxed and under gene names. mRNA expression of OLR1 gene was significantly different from others. (B-E) shows the expression patterns of 18 genes in heart, brain, gluteus and testis tissues from DSH1, DSH2 and DSH3, respectively, most of the gene expression patterns were similarity in the same tissues, differential genes were indicated by hollow triangle. (F-H) displays the expression profiles of 18 genes from DSH1, DSH2 and DSH3 in liver, heart, brain, gluteus and testis tissues, there were significantly different in five tissues from DSH1 and DSH3. However, there were similar in other four tissues in DSH2 except in liver tissue. (TIF) [file pone.0171442.s004.tif]

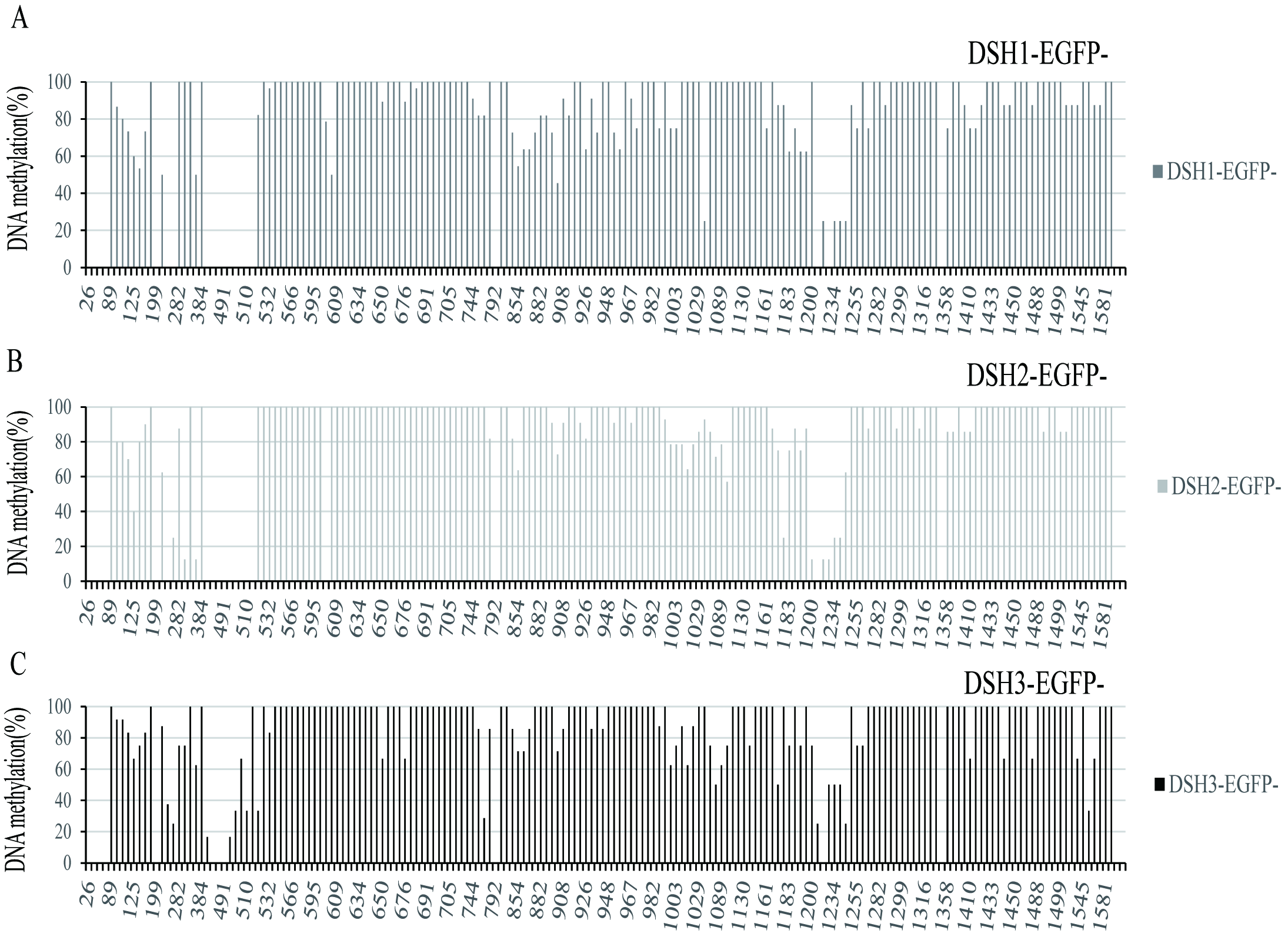

Supplement: S5 Fig — (A) DSH1. (B) DSH2. (C) DSH3. (TIF) [file pone.0171442.s005.tif]

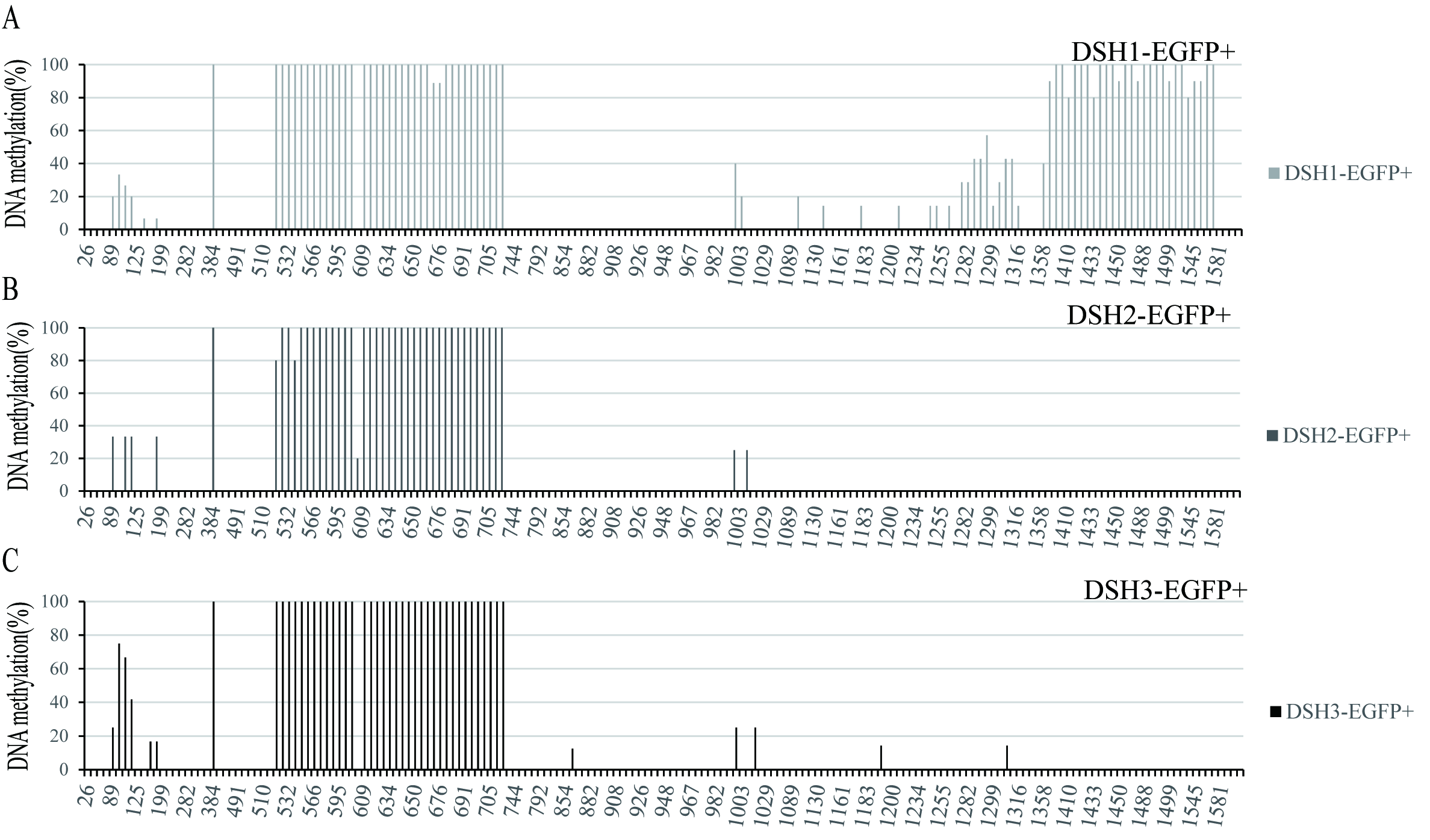

Supplement: S6 Fig — (A) DSH1. (B) DSH2. (C) DSH3. (TIF) [file pone.0171442.s006.tif]
